# Telomere-related gene risk model for prognosis and drug treatment efficiency prediction in kidney cancer

**DOI:** 10.3389/fimmu.2022.975057

**Published:** 2022-09-16

**Authors:** Song-Chao Li, Zhan-Kui Jia, Jin-Jian Yang, Xiang-hui Ning

**Affiliations:** Department of Urology, The First Affiliated Hospital of Zhengzhou University, Zhengzhou, China

**Keywords:** kidney cancer, telomere, prognosis, tumor mutation burden, immuno therapy

## Abstract

Kidney cancer is one of the most common urological cancers worldwide, and kidney renal clear cell cancer (KIRC) is the major histologic subtype. Our previous study found that von-Hippel Lindau (VHL) gene mutation, the dominant reason for sporadic KIRC and hereditary kidney cancer-VHL syndrome, could affect VHL disease-related cancers development by inducing telomere shortening. However, the prognosis role of telomere-related genes in kidney cancer has not been well discussed. In this study, we obtained the telomere-related genes (TRGs) from TelNet. We obtained the clinical information and TRGs expression status of kidney cancer patients in The Cancer Genome Atlas (TCGA) database, The International Cancer Genome Consortium (ICGC) database, and the Clinical Proteomic Tumor Analysis Consortium (CPTAC) database. Totally 353 TRGs were differential between tumor and normal tissues in the TCGA-KIRC dataset. The total TCGA cohort was divided into discovery and validation TCGA cohorts and then using univariate cox regression, lasso regression, and multivariate cox regression method to conduct data analysis sequentially, ten TRGs (ISG15, RFC2, TRIM15, NEK6, PRKCQ, ATP1A1, ELOVL3, TUBB2B, PLCL1, NR1H3) risk model had been constructed finally. The kidney patients in the high TRGs risk group represented a worse outcome in the discovery TCGA cohort (p<0.001), and the result was validated by these four cohorts (validation TCGA cohort, total TCGA cohort, ICGC cohort, and CPTAC cohort). In addition, the TRGs risk score is an independent risk factor for kidney cancer in all these five cohorts. And the high TRGs risk group correlated with worse immune subtypes and higher tumor mutation burden in cancer tissues. In addition, the high TRGs risk group might benefit from receiving immune checkpoint inhibitors and targeted therapy agents. Moreover, the proteins NEK6, RF2, and ISG15 were upregulated in tumors both at the RNA and protein levels, while PLCL1 and PRKCQ were downregulated. The other five genes may display the contrary expression status at the RNA and protein levels. In conclusion, we have constructed a telomere-related genes risk model for predicting the outcomes of kidney cancer patients, and the model may be helpful in selecting treatment agents for kidney cancer patients.

## Introduction

Kidney cancer is one of the most common urological cancers worldwide, and kidney renal clear cell cancer (KIRC) is the major histologic subtype (1, 2). The classic prognostic factors of kidney cancer are tumor features such as tumor stage, node stage, metastasis stage, and nuclear grade. Several models have been built to predict prognosis by combining the clinical features of cancer patients. These include the International Metastatic RCC Database Consortium (IMDC) risk model, UCLA Integrated Staging System (UISS), and Memorial Sloan-Kettering Cancer Center (MSKCC/Motzer) Score (3–6). Recently, owing to the development of sequencing technology, several molecular signatures have been identified to predict the overall survival of kidney cancer patients. These signatures show a robust predictive effect, and some of them could indicate the potential mechanism of cancer development (7, 8).

Telomeres are regions composed of repetitive TTAGGG DNA sequences and shelterin complex located at the end of chromosomes (9). Telomeres are essential for chromosome stability, and telomere length shortens following cell division and some disease statuses. In addition, abnormalities in the telomere might result in many diseases, such as dyskeratosis congenita, heart disease, mental health problems, and cancer (10, 11). Our previous study found that von-Hippel Lindau (VHL) gene mutation, the dominant reason for sporadic KIRC and hereditary kidney cancer- VHL syndrome, could affect VHL disease-related cancers development by inducing telomere shortening (12).

Moreover, many studies have been conducted to investigate the role of telomeres in the development and progression of cancers. Recent findings suggest that the shortening telomeres can affect the process of cancer development in two different ways. First, telomere shortening might play a tumor-suppressive role by arresting cell proliferation. On the other hand, telomere shortening could also result in extensive genome instability, which promotes cancer progression. In breast cancer, long telomere lengths correlated with a better prognosis (13). A meta-analysis indicated that short telomere length was associated with increased mortality risk and poor prognosis in cancer patients (14). In kidney cancer, telomere length in tumor cells was shorter than in normal kidney cells, but the prognostic role of telomere length remains controversial (15). However, shorter leukocyte telomere length in kidney cancer patients was an independent factor of worse outcomes, especially for stage I cancer patients (16).

Previous studies have focused on the telomere length in cancers and its role in the prognosis of cancers. There has been no study done to investigate telomere-related genes in the prognosis of cancers. Herein, we constructed a risk model using telomere-related genes to predict the prognosis of kidney cancer and then evaluated the potential role of this risk model in selecting treatment agents.

## Method

### Acquisition of data

Kidney cancer patients’ data files from The Cancer Genome Atlas (TCGA) dataset (TCGA-KIRC cohort), The International Cancer Genome Consortium (ICGC) database (RECA-EU cohort), and Clinical Proteomic Tumor Analysis Consortium (CPTAC) database (PDC000127) were downloaded and processed according to the operational processes of the public data provider. All the data in the TCGA-KIRC cohort were enrolled in this study to screen the differential expression genes. The analysis included the mRNA expression data and exact clinical features in all patients from these three datasets, including the survival time and tumor characteristics. The tumor mutation burden (TMB) data of the TCGA-KIRC cohorts were also downloaded and processed. In addition, the protein expression status of the CPTAC cohort and the immunohistochemical (IHC) data of renal cancer in the Human Protein Atlas (HPA) database were also acquired. In addition, the telomere-related genes were obtained from http://www.cancertelsys.org/telnet/ (17).

### Screening and analysis of differential telomere-related genes

The RNA sequence profiles of 533 kidney cancer tissues and 72 adjacent normal kidney tissues were used for screening the differential genes using the limma package (18). Telomere-related genes in all the differential genes were selected and enrolled in weighted gene co-expression network analysis (WGCNA), which was conducted using the WGCNA R package (19). The most differentially expressed telomere-related genes between the tumor and the adjacent normal kidney tissues in the WCGNA results were included in the construction of the prognosis model.

### Construction and verification of telomere-related genes risk model

The TCGA-KIRC cohort was randomly divided into the discovery TCGA cohort and the validation cohort in a 1:1 ratio. The discovery TCGA cohort was analyzed sequentially through univariate cox regression, Lass regression, and multivariate cox regression. Subsequently, the risk model was constructed based on the genes’ expression value and the coefficient, which were acquired in the multivariate cox regression using the Akaike Information Criterion (AIC) method. The risk score of each patient was calculated in these five cohorts: discovery TCGA cohort, validation TCGA cohort, total TCGA cohort, ICGC cohort, and CPTAC cohort. Each cohort was divided into two groups, the high-risk group, and the low-risk group, according to the median risk score, and the prognosis of each risk group was examined using the log-rank test. The risk score was also evaluated using univariate and multivariate cox regression to determine its role in predicting the overall survival of kidney cancer patients in the five cohorts. In addition, the receiver operating characteristic (ROC) curve was used to check the accuracy of the risk model in predicting the prognosis. Finally, a nomogram was constructed to predict the 1-, 3-, and 5-year survival rates using the risk score based on the total TCGA cohort.

### Immune features and immune subtype analysis

The infiltration of the different types of immune cells in the kidney cancer tumor microenvironment was assessed by the specific genes’ expression value using CIBERSORT iterated 1000 times (https://cibersort.stanford.edu/) (20). The immune subtypes of pan-cancers in the TCGA database have been described in a previous study, and the immune subtypes of KIRC patients were obtained (21). Additionally, TIDE (Tumor ImmuneDysfunction and Exclusion) score was calculated online following the instructions (https://tide.dfci.harvard.edu/) (22).

### IC50 prediction of the different targeted therapy agents

The targeted drugs’ half-maximal inhibitory concentrations (IC50) were predicted using the gene expression level to reflect the treatment sensitivity. This was done using the R package named “pRRophetic” (23, 24).

### Cell culture, RNA Extraction and quantitative real-time PCR

Totally seven cell lines, including a normal human renal proximal tubular cell line (HK2) and six RCC cell lines (Caki-1, 769-P, OSRC-2, ACHN, 786-O, A498) were used to investigate the telomere-related gene expression status. Among these cell lines, 769-P, A498, 786-O, and OSRC-2 were cultured in RPMI 1640 medium supplemented with 10% fetal bovine serum. While HK2, Caki-1, and ACHN were cultured in DMEM medium with 10% fetal bovine serum. TRIZOL reagent (Invitrogen, USA) was used to extract the total RNA, and the reverse transcription reactions were carried out using a qPCR RT Kit (TOYOBO Life Science, Shanghai, China). Expression levels of telomere-related genes were detected by qRT-PCR). The human beta-actin gene was used as a reference gene. The primers were shown in **Table 1** (25). The experiment was conducted on a Bio-Rad S1000 machine and using an SYBR Green RT-PCR Master Mix reagent (TOYOBO). TRGs’ Relative expression value was computed using the 2^−ΔΔCt^ method and normalized with the beta-actin (26).

**Table 1:** The primers used in qRT-PCR of the TRG risk model genes.

Genes	Forward Primer sequence	Reverse Primer sequence
ISG15	CGCAGATCACCCAGAAGATCG	TTCGTCGCATTTGTCCACCA
RFC2	GTGAGCAGGCTAGAGGTCTTT	TGAGTTCCAACATGGCATCTTTG
TRIM15	AGGCCATTTCTCCTGACCTTG	CCGGGTGTACCTCACTGAC
NEK6	GCTCGGTGACCTTGGTCTG	CGGACTTGAAGTTGTAGCCGT
PRKCQ	GCAAAAACGTGGACCTCATCT	CAAAGAAGCCTTCCGTCTCAAA
ATP1A1	CTGTGGATTGGAGCGATTCTT	TTACAACGGCTGATAGCACCA
ELOVL3	TGGGGCATTATGGGGACTGT	AGGACCAGAATTTGACTGTGGA
TUBB2B	GGCACGATGGATTCGGTTAGG	ACACGAAATTGTCTGGTCTGAAG
PLCL1	AAAGTCCGGCCAAATTCTCG	TTTCCGTGTTTTTCCCCAGTC
NR1H3	TCTGGAGACATCTCGGAGGTA	GGCCCTGGAGAACTCGAAG

### Protein expression analysis and statistical analysis

The expression of proteins encoded by the genes included in the telomere-related genes model in the CPTAC cohort was compared using the limma package. In addition, the protein expression status of renal cancer and kidney tissues in the HPA database was accessed and analyzed using the “HPAanalyze” package (27). Continuous variables were compared by independent t-test, while the categorical variables were analyzed using the chi-square test. A P-value < 0.05 (two sides) was considered statistically significant.

## Results

### Analysis of the differential telomere-related genes

**Figure 1:**
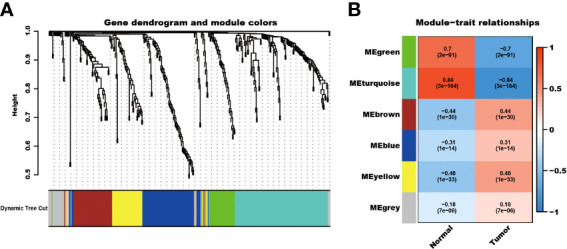
WCGNA analysis of differential expressed telomere-related genes. The differential expressed telomere-related genes were clustered into six modules **(A)**, and the ME-turquoise module shows the most significant difference **(B)**.

The detailed characteristics of the three cohorts are summarized in the **Supplementary Table**. A total of 2086 telomere-related genes were obtained from TelNet. Among these, 353 genes were differentially expressed between kidney cancer and adjacent normal kidney tissues. In particular, 234 genes were upregulated, while 119 were downregulated in the tumor tissues (**Supplementary Figure 1**). In addition, these differential telomere-related genes were analyzed using the WCGNA method. The results showed that these genes were clustered into six models, MEgreen, MEturquoise, ME brown, MEblue, MEyellow, and MEgrey modules. Among these modules, the ME-turquoise module, composed of 118 genes, displayed the most significant difference between normal kidney and kidney cancer tissues (R^2 =^ 0.84, P<0.001, **Figure 1**).

### Telomere-related genes risk model could predict the prognosis of kidney cancer patients

**Figure 2:**
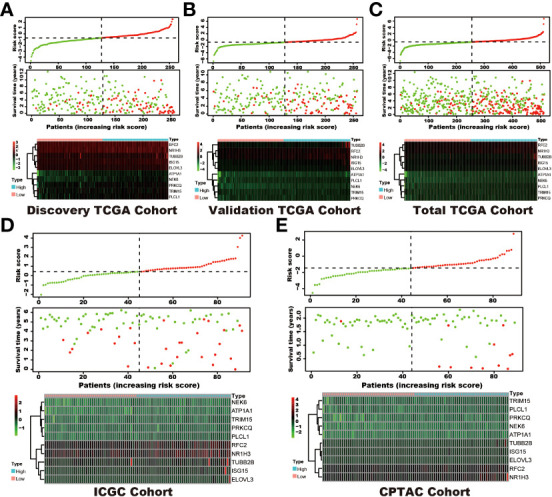
The distribution of survival status and TRGs risk score in these six cohorts. The patients were ordered according to the TRGs risk score, shown in the up panel, and the survival status of each patient with a different risk score was shown in the middle panel. The TRGs risk model gene expression value has presented in the lower panel. **(A)** discovery TCGA cohort; **(B)** validation TCGA cohort; **(C)** total TCGA cohort; **(D)** ICGC cohort; **(E)** CPTAC cohort.

**Figure 3:**
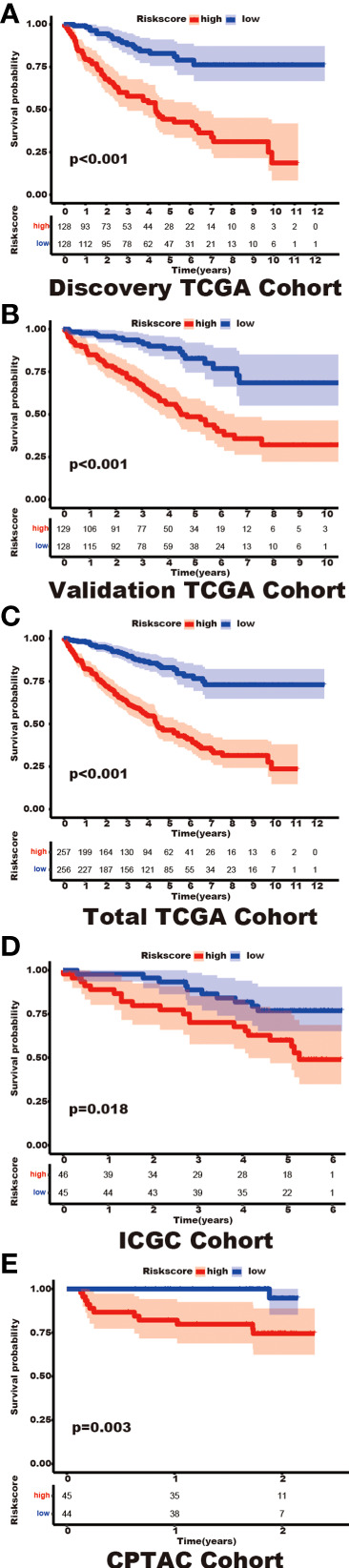
The differential prognosis in patients with different TRGs risk groups. Log-rank methods had compared the survival status in different TRGs risk groups, the patients in the high TRGs risk group presented worse outcomes in these five cohorts. **(A)** discovery TCGA cohort; **(B)** validation TCGA cohort; **(C)** total TCGA cohort; **(D)** ICGC cohort; **(E)** CPTAC cohort.

**Figure 4:**
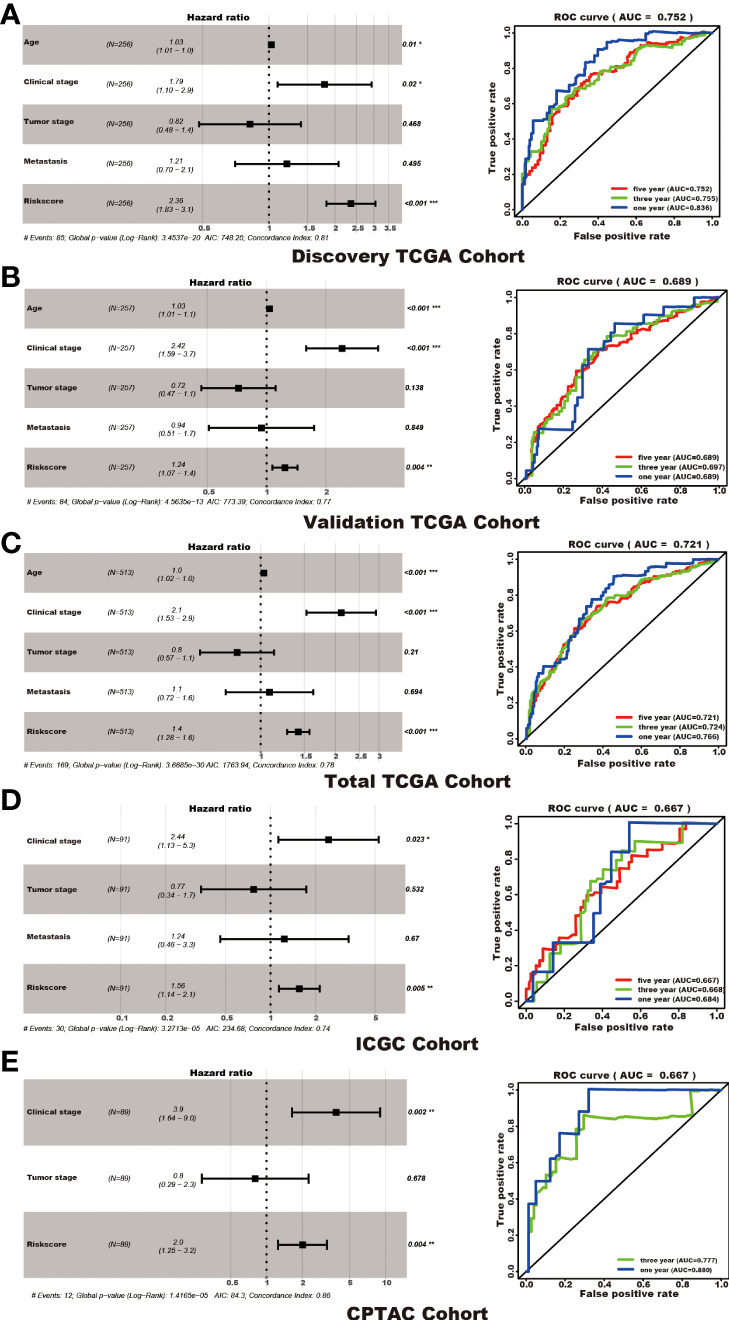
TRGs risk model as an independent prognosis factor in these five cohorts. TRGs risk score, clinical stage, and age were the independent factors in the discovery TCGA cohort **(A)**, validation TCGA cohort **(B)**, and total TCGA cohort **(C)**. Clinical stage and TRGs risk score were the independent factors in the ICGC cohort **(D)** and CPTAC cohort **(E)**. The ROC curve and AUC value were also shown in each cohort. (*represents p<0.01,**represents p<0.01, *** represents p<0.001).

**Figure 5:**
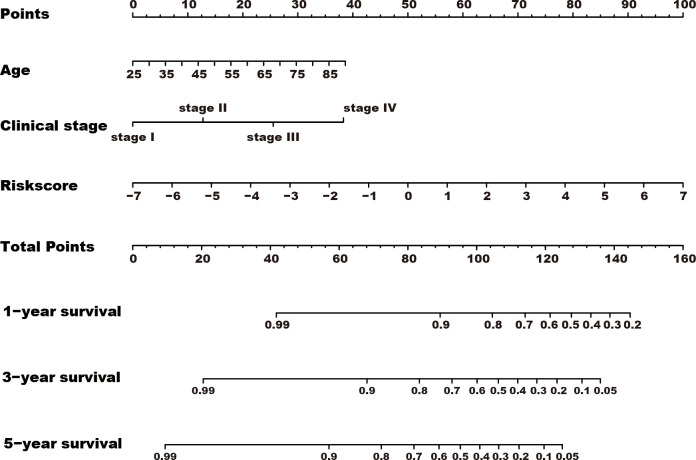
A nomogram was constructed for predicting the overall survival of kidney cancer patients. TRGs risk score, clinical stage, and age composed the nomogram using the total TCGA cohort, and the risk score has the most contribution to the total points.

Among the 353 differential expression genes, 47 genes correlated with kidney cancer patients’ overall survival in the discovery TCGA cohort. Then 19 genes in these 47 genes were finally screened by Lasso regression. Finally, ten telomere-related genes (ISG15, RFC2, TRIM15, NEK6, PRKCQ, ATP1A1, ELOVL3, TUBB2B, PLCL1, NR1H3) in these 19 genes were identified as independent risk factors through the multivariate cox regression and were used to format the risk model, telomere related genes (TRGs) risk model. And the TRGs risk score formula was as follows: risk score=0.005134398*ISG15 +0.047733021*RFC2-0.071026696*TRIM15-0.0203981*NEK6-0.133649782*PRKCQ-0.007396692*A TP1A1+0.700714514*ELOVL3+0.206037577*TUBB2B-0.148322187*PLCL1+0.070770091*NR1H3. The risk score of patients in five cohorts was computed, and the patients in each cohort were divided into a high and low-risk group according to each cohort’s median risk score value. The patients in the high-risk group represent the worse outcomes in all these five cohorts, discovery TCGA cohort (p<0.001), validation TCGA cohort (p<0.001), total TCGA cohort (p<0.001), ICGC cohort (p=0.018) and CPTAC cohort (p=0.003) (**Figures 2**, **3**). In addition, the TRGs risk score proved to be an independent prognostic factor for kidney cancer patients in these five cohorts (**Figure 4** and **Table 2**). Moreover, a nomogram, which consisted of patients’ age, clinical stage, and TRGs risk score, was constructed to individually predict kidney cancer patients’ overall survival (**Figure 5**).

**Table 2:** Univariate and Multivariate Cox regression reveals TRGs risk score is an independent risk factor in kidney cancer.

	Variables	Univariate cox regression		Variables	Multivariate cox regression	
		HR (95%CI)	p-value		HR (95%CI)	p-value
**Discovery TCGA cohort**	Age	1.031 (1.012-1.051)	**0.002**	Age	1.026 (1.006-1.047)	**0.010**
	Gender	0.862 (0.552-1.346)	0.514			
	Clinical stage	1.952 (1.601-2.38)	**<0.001**	Clinical stage	1.792 (1.095-2.932)	**0.020**
	T stage	2.047 (1.592-2.633)	**<0.001**	T stage	0.821 (0.481-1.399)	0.468
	M stage	2.086 (1.531-2.842)	**<0.001**	M stage	1.207 (0.703-2.075)	0.495
	N stage	0.848 (0.683-1.053)	0.136			
	Riskscore	2.718 (2.133-3.464)	**<0.001**	Riskscore	2.362 (1.827-3.054)	**<0.001**
**Validation TCGA cohort**	Age	1.028 (1.009-1.046)	**0.003**	Age	1.034 (1.015-1.054)	**0.001**
	Gender	1.309 (0.84-2.039)	0.235			
	Clinical stage	1.871 (1.559-2.246)	**<0.001**	Clinical stage	2.416 (1.587-3.676)	**<0.001**
	T stage	1.86 (1.484-2.331)	**<0.001**	T stage	0.72 (0.466-1.111)	0.138
	M stage	2.291 (1.605-3.27)	**<0.001**	M stage	0.942 (0.51-1.741)	0.849
	N stage	0.966 (0.778-1.2)	0.756			
	Riskscore	1.238 (1.1-1.394)	**<0.001**	Riskscore	1.239 (1.069-1.435)	**0.004**
**Total TCGA cohort**	Age	1.029 (1.016-1.043)	**<0.001**	Age	1.031 (1.016-1.045)	**<0.001**
	Gender	1.049 (0.767-1.437)	0.764			
	Clinical stage	1.895 (1.659-2.166)	**<0.001**	Clinical stage	2.114 (1.53-2.92)	**<0.001**
	T stage	1.919 (1.626-2.264)	**<0.001**	T stage	0.803 (0.569-1.131)	0.210
	M stage	2.179 (1.728-2.748)	**<0.001**	M stage	1.085 (0.722-1.63)	0.694
	N stage	0.905 (0.777-1.054)	0.200			
	Riskscore	1.392 (1.28-1.515)	**<0.001**	Riskscore	1.419 (1.28-1.573)	**<0.001**
**ICGC cohort**	Age	1.031 (0.993-1.071)	0.109			
	Gender	0.939 (0.456-1.933)	0.863			
	Clinical stage	2.094 (1.515-2.896)	**<0.001**	Clinical stage	2.444 (1.132-5.276)	**0.023**
	T stage	1.989 (1.402-2.821)	**<0.001**	T stage	0.772 (0.344-1.737)	0.532
	M stage	2.522 (1.394-4.562)	**0.002**	M stage	1.239 (0.462-3.326)	0.67
	N stage	1.162 (0.696-1.938)	0.566			
	Riskscore	1.492 (1.127-1.975)	**0.005**	Riskscore	1.558 (1.14-2.129)	**0.005**
**CPTAC cohort**	Age	1.014 (0.964-1.065)	0.596			
	Gender	1.318 (0.356-4.873)	0.679			
	Clinical stage	3.853 (1.819-8.164)	**<0.001**	Clinical stage	3.851 (1.642-9.031)	**0.002**
	T stage	2.894 (1.287-6.506)	**0.01**	T stage	0.803 (0.286-2.258)	0.678
	M stage	0.781 (0.429-1.423)	0.42			
	N stage	0.331 (0.1-1.102)	0.072			
	Riskscore	1.972 (1.369-2.841)	**<0.001**	Riskscore	2.01 (1.251-3.23)	**0.004**

The bold values implied the correspondence variable was shown significant difference in Univariates or Multivariate Cox regression analysis.

### The patients with high-risk scores present high TMB

**Figure 6:**
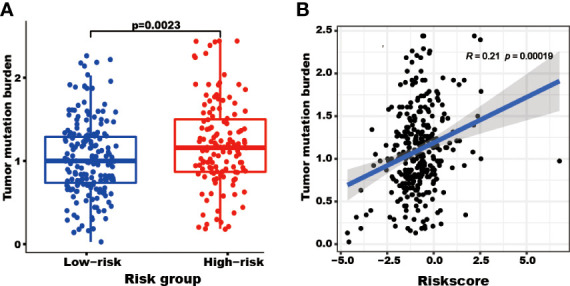
The tumor mutation burden (TMB) correlated with the TRGs risk score and groups. The TMB of patients in the high TRGs risk group (red points) was higher than those in the low TRGs group (blue points). **(A)** The patients’ TMB positively correlated with TRGs risk score (p=0.00019). **(B)**.

The patients in the high-risk group showed a higher TMB (P=0.0023) than those in the low-risk group (**Figure 6A**). Furthermore, the risk score correlated with the TMB (R=0.21, P=0.00019, **Figure 6B**).

### The patients in different risk groups show different immune status

**Figure 7:**
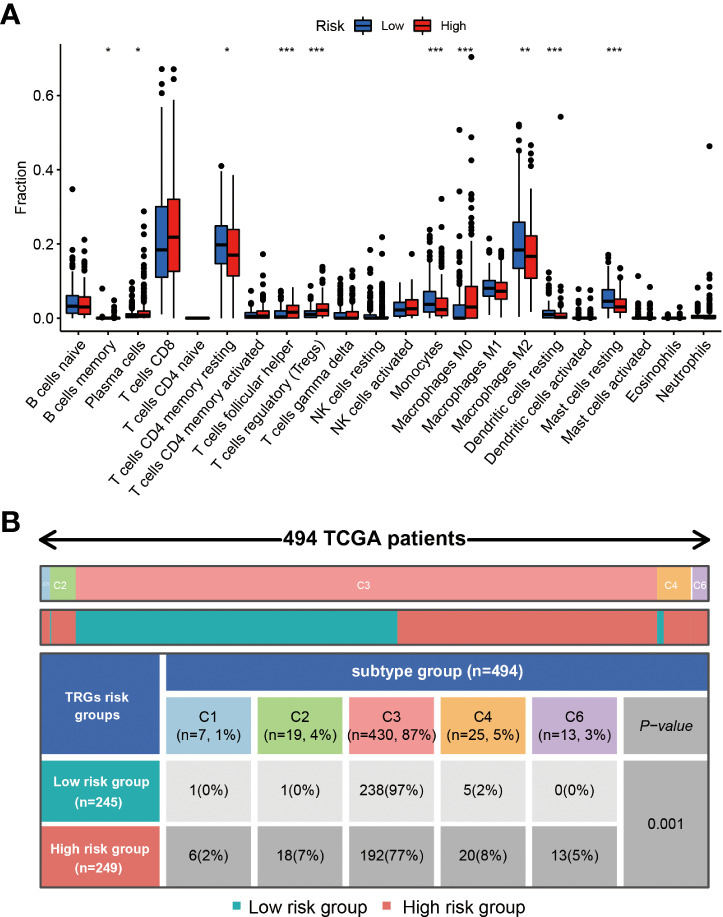
The immune cells and subtypes of the TRGs risk group. The immune cells distribution portion in the cancer tissues with different TRGs risk group patients. (*represents p<0.01, **represents p<0.01, *** represents p<0.001). **(A)** Four hundred of ninety-four patients in the total TCGA cohort has the immune subtype results, no C5 subtype in these patients, and C3 is the most common subtype in the two TRGs risk group. **(B)**.

Different types of immune cells exhibited different infiltration rates in the tumor microenvironment between the high and low-risk groups. Plasma cells, T cells follicular helper, T cells regulatory (Tregs), and macrophages M0 had a higher rate of infiltration in the high-risk group than in the low-risk group. T cells CD4 memory resting, monocytes, macrophages M2, dendritic cells resting, and mast cells resting showed less infiltration in the high-risk group than in the low-risk group (**Figure 7A**).

Previous studies have clustered the tumors in the TCGA database into six subtypes according to the immune status: C1 (wound healing), C2 (IFN-g dominant), C3 (inflammatory), C4 (lymphocyte depleted), C5 (immunologically quiet), and C6 (TGF-b dominant) (21). The immune subtype analysis results indicated the patients in the high-risk group had a higher proportion of C1(2%), C2(7%), C4(8%), and C6(5%) subtype than the patients in the low-risk group, and a lower proportion of C3(77%) subtype than the patients in the low-risk group (97%) (**Figure 7B**).

### Tumor-related genes risk model could be used in choosing a treatment strategy

**Figure 8:**
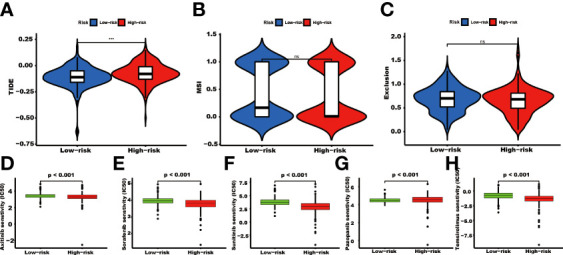
The TIDE score and targeted agents’ treatment sensitivity. Tumor tissues in the high TRGs risk group present the high TIDE score **(A)**, and the MSI and Expulsion score has no significant difference in these two groups (*** represents p<0.001, ns represents no significant difference). **(B, C)**. The treatment response of targeted agents, axitinib **(D)**, sorafenib **(E)**, sunitinib **(F)**, pazopanib **(G)**, and temsirolimus **(H)**.

The TIDE score was significantly higher in the high-risk group than in the low-risk group (P<0.001, **Figure 8A**). There were no significant differences in the MSI and exclusion score (**Figures 8B, C**). The IC50 values of Axitinib, Sorafenib, Sunitinib, and Temsirolimus in the high-risk group were lower than in the low-risk group. In contrast, the IC50 value of Pazopanib was higher in the high-risk group than in the low-risk group (**Figures 8D–H**).

### RNA and protein expression status of the tumor-related genes used in the model

**Figure 9:**
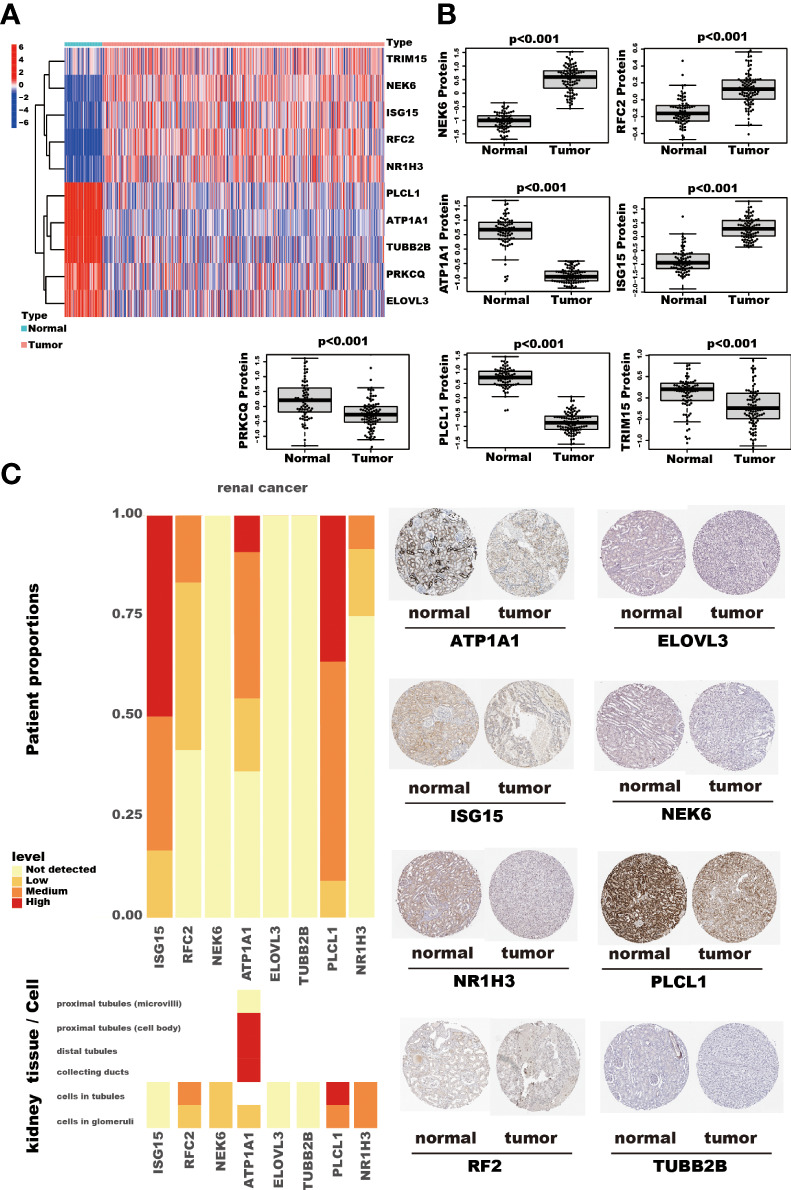
The RNA and protein expression of TRGs model genes. The heatmap shows these ten TRGs model gene expression in kidney cancer and adjacent normal kidney tissues. **(A)** The seven proteins expression, assessed by Tandem Mass Tag (TMT) 10 method, compares the kidney cancer and adjacent normal kidney tissues in the CPTAC cohort. The thin lines represent the maximum and minimum values, and the thick line represents the median value. **(B)** The detection of eight proteins by IHC in the HPA database. **(C)**.

**Figure 10:**
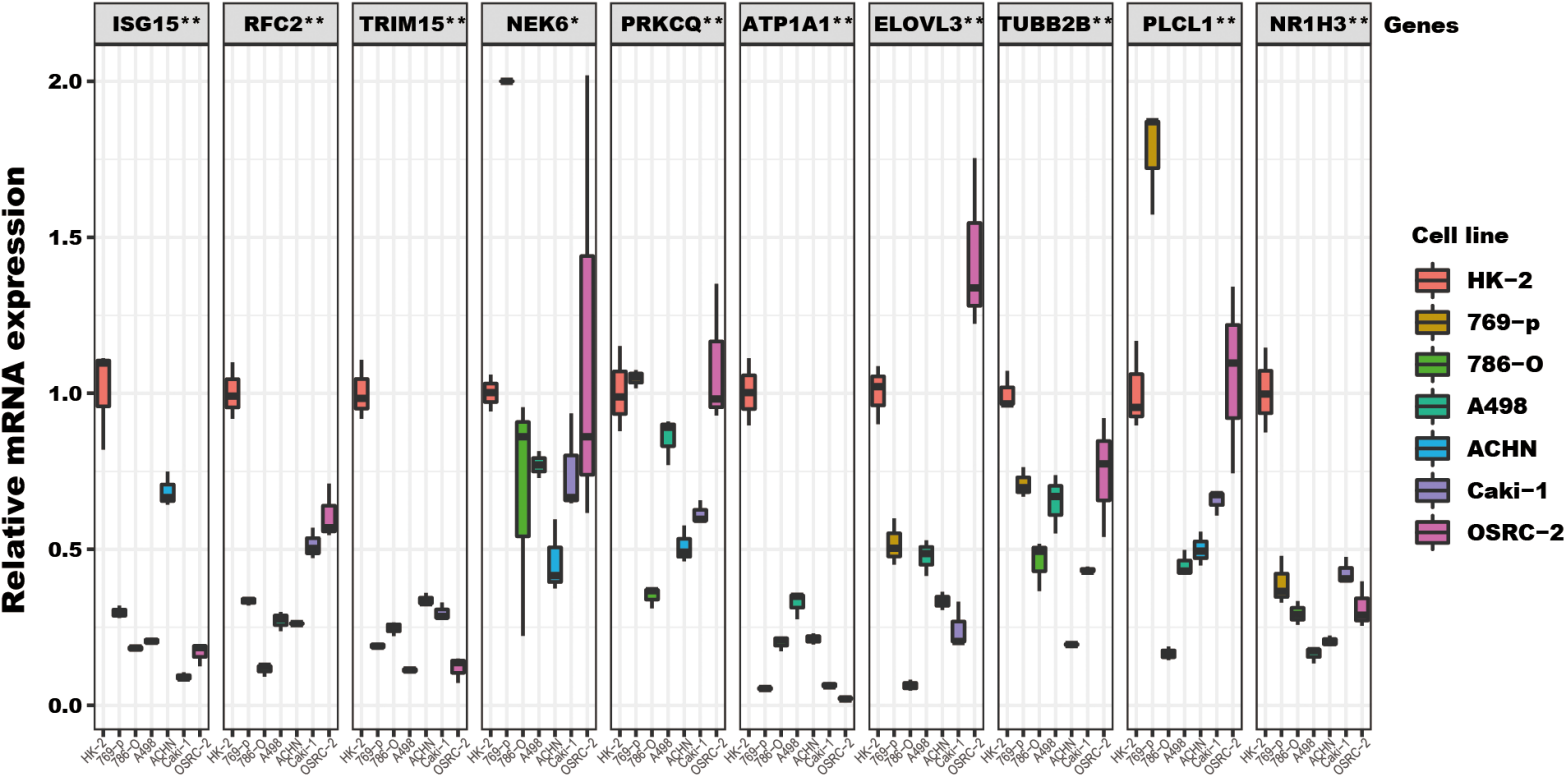
The TRGs model genes’ relative expression status in RCC cell lines. These 10 genes’ relative expression was assessed by qRT-PCR in HK2 and six RCC cell lines (Caki-1, 769-P, OSRC-2, ACHN, 786-O, A498). All these genes’ expressions in RCC cell lines were compared to their expressions in the HK2 cell line. The NEK6 gene expression in 769-P was set to 2, and the true value was more than 2 (**Supplementary File**). The headline of this figure represents the TRGs model genes name. (*represents p<0.01, **represents p<0.01).

Among the ten tumor-related genes used in the risk model, TRIM15, NEK6, ISG15, RFC2, and NR1H3 were upregulated. At the same time, PLCL1, ATP1A1, TUBB2B, PRKCQ, and ELOVL3 were downregulated in tumors in the TCGA-KIRC dataset at the transcription level (**Figure 9A**). At the protein level, seven proteins were notable in the tumors of the CPTAC cohort. ATP1A1, ISG15, NEK6, and RFC2 were significantly upregulated, while PRKCQ, PLCL1, and TRIM15 were downregulated (**Figure 9B**). In addition, eight proteins had IHC results in the HPA database. Though the exact differences between normal kidney tissue and tumors cannot be thoroughly evaluated, the primary data showed that ISG15 might be upregulated in tumors. At the same time, NEK6 and NR1H3 might be downregulated in tumor tissues. No significant differences were found in the expression of RFC2, ATP1A1, ELOVL3, TUBB2B, and PLCL1 (**Figure 9C**). In addition, these genes’ relative expression status was assessed in RCC cell lines by qRT-PCR, and the results show a significant difference in these cell lines (**Figure 10**).

## Discussion

Telomeres play an essential role in the development of kidney cancer. To the best of our knowledge, this study is the first to assess the role of telomere-related genes in the prognosis of renal cancer. We established a prognostic model based on telomere-related genes using public databases. We found that this model can serve as a basis for selecting therapeutic drugs for renal cancer.

Previous studies show the varied prognosis of the tumors in different immune subtypes, the C3 subtype presented with the most favorable outcome, while the C6 and C4 subtypes had the worst prognosis. Our results suggested that the telomere-related genes risk model group was significantly correlated with the immune subtypes. The high-risk group had a higher proportion of C1, C2, C4, and C6 subtypes and a lower proportion of the C3 subtype than the low-risk group. The immune subtype analysis indicated that the patients in the high-risk group might have had a poor prognosis due to the higher portion of subtypes (C1+C2+C4+C6, 23%) with a worse outcome than in the low-risk group (3%). In addition, the high-risk group showed a high TIDE score, indicating that the high-risk group patients may be more likely to experience an immune escape. Overall, the results suggest that the differences in prognosis between the high and low-risk groups might be partly due to the different immune statuses of the patients.

High TMB has been identified as a biomarker to predict the potential benefit of immune checkpoint blockade (ICB) therapy. Our study showed that the high-risk group was associated with a high TMB (28). The drug sensitivity prediction analysis results showed that the high-risk group patients might be more sensitive to Axitinib, Sorafenib, Sunitinib, and Temsirolimus treatment. In contrast, those in the low-risk group might benefit from Pazopanib treatment. Taken together, the patients with a high-risk score might be more suitable candidates to receive immune checkpoint inhibitors and targeted therapy agents. In contrast, the patients with a low-risk score had a limited selection of treatment.

The genes in the telomere-related genes risk model play varying roles in disease. ATP1A1 encodes the α-1 isoform of the familiar Na^+^/K^+^ATPase. Studies have found that ATP1A1 mutations could cause aldosterone-producing adenoma (APA). Inhibiting ATP1A1 expression in pancreatic ductal adenocarcinoma (PDAC) cells can suppress the tumor invasion. In kidney cancer, the promoter methylation rate of ATP1A1 was about 15.8% (29–32). Replication Factor C Subunit 2 (RFC2) gene encodes subunit 2 of the RFC complex, a primer recognition factor of DNA polymerase. Studies have shown that RFC2 could regulate the cell cycle and DNA replication process to promote liver cancer development and could act as an oncogene in the progression of lower-grade gliomas (33, 34). Tripartite Motif Containing 15 (TRIM15) encodes a member of the Tripartite motif family, which could exhibit E3 ubiquitin ligase activity. TRIM15 could regulate the Wnt/β-catenin signaling pathway and Keap1-Nrf2 pathway and mediate the ubiquitination of APOA1 and ERK to promote the development and progression of cancers such as non-small cell lung cancer, esophageal squamous cell carcinoma, and pancreatic cancer (35–37). NIMA Related Kinase 6 (NEK6) encodes a kinase, which plays multiple roles in the tumor, including suppression of tumor cell senescence and facilitation of breast cancer cell proliferation. Moreover, NEK6 could participate in the development of castration resistance in prostate cancer. In kidney cancer, studies revealed that miR-141-3p regulated NEK6 to influence cell proliferation, migration, and apoptosis of tumor cells (38–41). Protein Kinase C Theta (PRKCQ) encodes a serine/threonine kinase. It can regulate the immune system by controlling T cells’ activation, survival, and differentiation. PRKCQ affects different processes in cancers, including tumor cell proliferation, migration, and invasion (42–44). IFN-stimulated gene factor 15 (ISG15) encodes a protein induced by type I IFNs, and it is a ubiquitin-like protein. ISG15 exists in two forms *in vivo*: conjugated to other proteins or free protein. ISG15 and its conjugation affect the progression and treatment response in different cancers. In kidney cancer, ISG15 has been investigated as a novel protein adjuvant in vaccines (45–48). ELOVL Fatty Acid Elongase 3 (ELOVL3) encodes a long-chain fatty acid elongase protein expressed in the liver and brown adipose tissues. Previous studies have shown that ELOVL3 could predict the prognosis of cancers (49–51). Tubulin Beta 2B Class IIb (TUBB2B) encodes a beta isoform of tubulin. The protein could bind GTP and is the major component of microtubules. TUBB2B has been shown to participate in the construction of a prognostic model of kidney cancer (52). Phospholipase C Like 1 (PLCL1) encodes a protein that was predicted to enable phospholipase C activity. PLCL1 was downregulated in kidney cancer tissues and correlated with a poor prognosis. In addition, PLCL1 could repress the progression of kidney cancer through UCP1-mediated lipid browning (53). Nuclear Receptor Subfamily 1 Group H Member 3 (NR1H3), also known as LXR-A, encodes a protein that belongs to the nuclear receptor superfamily. Elevated LXRα expression correlates with a high tumor stage, histologic grade, and pathologic stage of ccRCC, and this could regulate ccRCC cell migration and invasion (54, 55). In our study, NEK6, RF2, and ISG15 were upregulated in tumors both at the RNA and protein levels, while PLCL1 and PRKCQ were downregulated. The other five genes may display the contrary expression status at the RNA and protein levels. The different expression status reflects the different transcriptional, and post-transcriptional mechanisms of these genes in kidney cancer cells, the detailed mechanism and role of these risk model genes should be investigated further.

Our study has some limitations. Though we used three databases to construct and validate this ten genes TRGs risk model, we cannot carry out a single trial to verify our findings due to the long translation and follow-up circle and the high cost (56, 57). In addition, as our results were based on the transcriptomics profile, which limited the clinical application and promotion of the TRGs risk model, a further simple and easy method should be developed. And the functions of the TRGs risk model genes in kidney cancer should be clarified by more basic experiments in further.

In this study, we have constructed a telomere-related genes risk model using the TCGA dataset and carefully verified the model in two datasets (CPTAC and ICGC). We have discussed the protein and RNA expression status of these genes in kidney cancer; however, there were no experiments to validate our findings. The model we have constructed may be helpful in the selection of treatment agents for kidney cancer patients.

## Data availability statement

The datasets presented in this study can be found in online repositories. The names of the repository/repositories and accession number(s) can be found in the article/**Supplementary Material**.

## Ethics statement

Ethical review and approval was not required for the study on human participants in accordance with the local legislation and institutional requirements. Written informed consent for participation was not required for this study in accordance with the national legislation and the institutional requirements.

## Author contributions

Conceived and designed the experiment: X-HN, S-CL. Performed the experiments and analyzed the data: X-HN, S-CL, Z-KJ. Interpretation of the findings: X-HN, S-CL, Z-KJ and J-JY. All authors contributed to the article and approved the submitted version.

## Funding

This work was supported by the Postdoctoral Research Grant in Henan Province (grant number 1901004), the Henan Science and Technology Research Program (grant number 2018020142), and The Natural Science Foundation of Henan Province (212300410265). The funders had no role in study design, data collection and analysis, decision to publish, or manuscript preparation.

## Acknowledgments

The results shown here are in whole or part, based upon data generated by the TCGA Research Network (https://www.cancer.gov/tcga), ICGC database (https://dcc.icgc.org/), and CPTAC (https://proteomic.datacommons.cancer.gov/pdc/study/PDC000127).

## Conflict of interest

The authors declare that the research was conducted in the absence of any commercial or financial relationships that could be construed as a potential conflict of interest.

## Publisher’s note

All claims expressed in this article are solely those of the authors and do not necessarily represent those of their affiliated organizations, or those of the publisher, the editors and the reviewers. Any product that may be evaluated in this article, or claim that may be made by its manufacturer, is not guaranteed or endorsed by the publisher.
