# Supplementary figures and images for: Telomere-related gene risk model for prognosis and drug treatment efficiency prediction in kidney cancer
Source: Front Immunol. 2022 Sep 16;13:975057. doi: 10.3389/fimmu.2022.975057 (PMC9523360; doi:10.3389/fimmu.2022.975057)

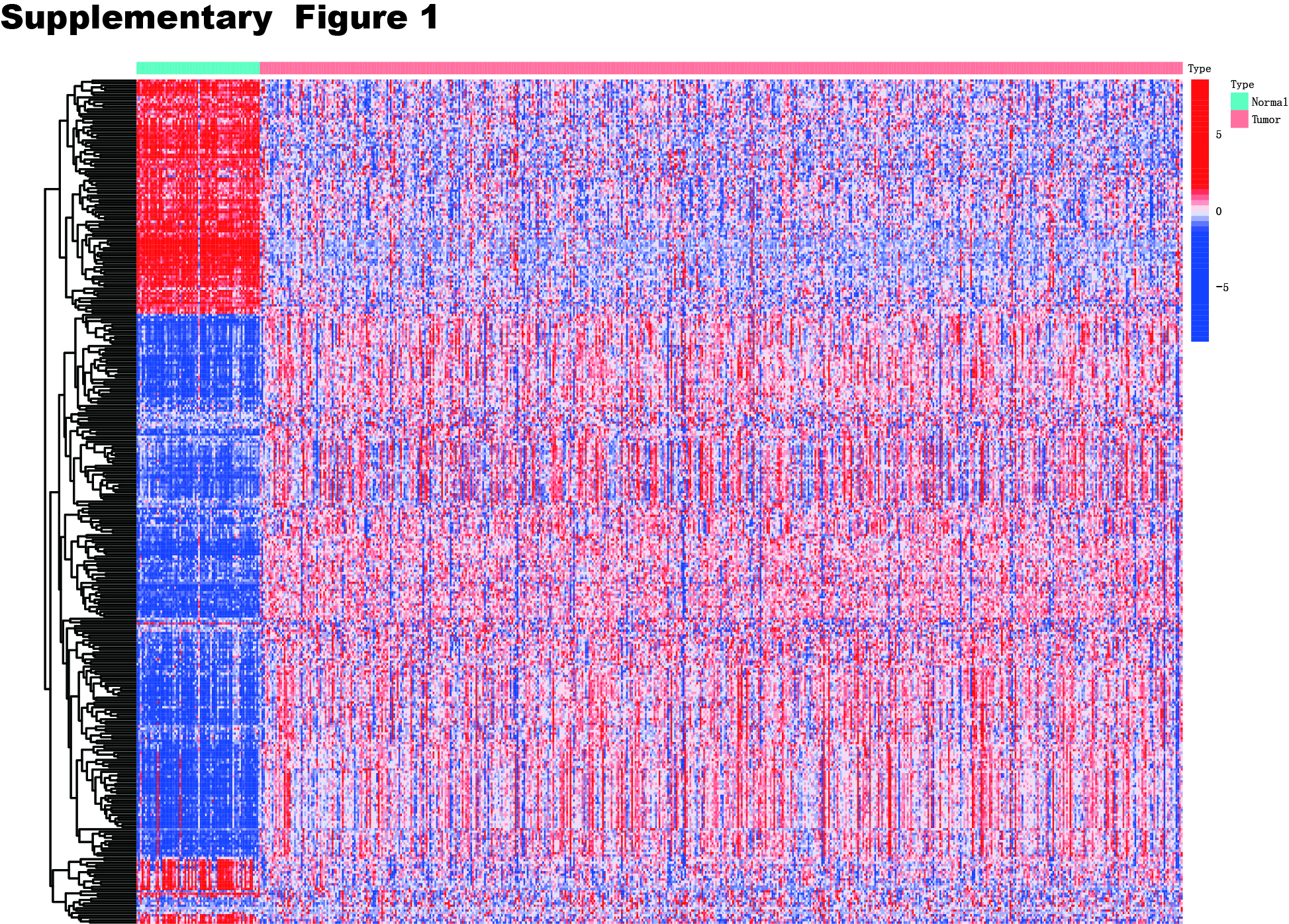

Supplement: Supplementary file 2 [file Image_1.jpeg]

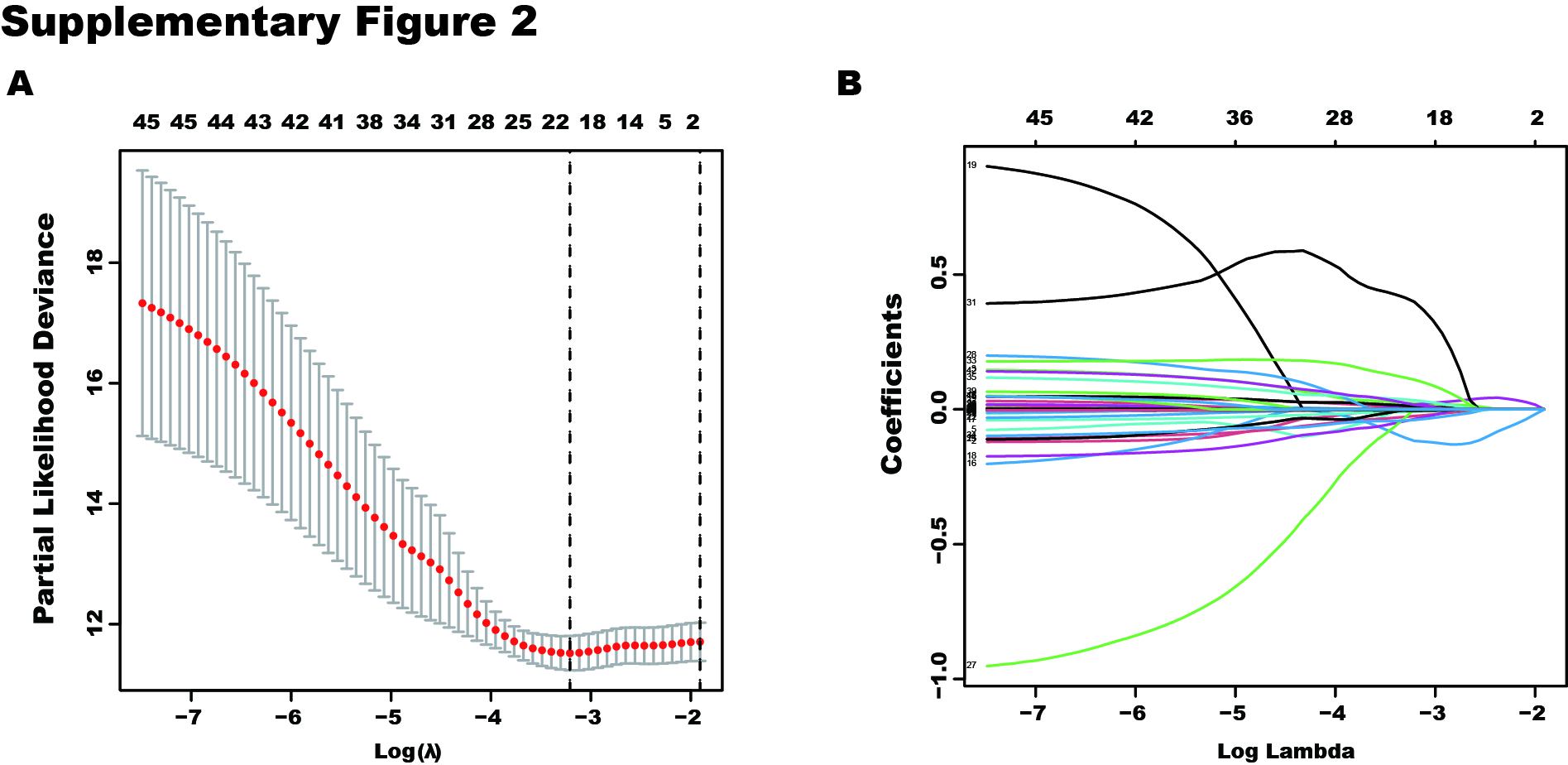

Supplement: Supplementary file 3 [file Image_2.jpeg]
