# Supplementary material for: Telomere-related gene risk model for prognosis and drug treatment efficiency prediction in kidney cancer
Source: Front Immunol. 2022 Sep 16;13:975057. doi: 10.3389/fimmu.2022.975057 (PMC9523360; doi:10.3389/fimmu.2022.975057)
Supplement: Supplementary file 4 [file Table_1.docx]

| **Variables** | **Discovery TCGA cohort (n=256)** | **Validation TCGA cohort (n=257)** | **Total TCGA cohort (n=513)** | **ICGC cohort (n=91)** | **CPTAC cohort (n=89)** |
| --- | --- | --- | --- | --- | --- |
| **Age (years)** | 60(26-86) | 61(32-88) | 60(26-88) | 60(35-83) | 60(30-89) |
| **Gender** |  |  |  |  |  |
| **Male** | 164(64.1%） | 172(66.9%） | 336(65.5%） | 52(57.1%） | 69(77.5%） |
| **Female** | 92(35.9%） | 85(33.1%） | 177(34.5%） | 39(42.9%） | 20(22.5%） |
| **Clinical stage** |  |  |  |  |  |
| **Stage I** | 115(44.9%) | 140(54.5%) | 255(49.8%) | 53(58.2%) | 42(47.2%) |
| **Stage II** | 26(10.1%) | 28(10.9%) | 54(10.5%) | 13(14.3%) | 10(11.2%) |
| **Stage III** | 69(27.0%) | 53(20.6%) | 122(23.8%) | 16(17.6%) | 28(31.5%) |
| **Stage IV** | 46(18.0%) | 36(14.0%) | 82(15.9%) | 9(9.9%) | 9(10.1%) |
| **T stage** |  |  |  |  |  |
| **T1** | 117(45.7%) | 144(56.0%) | 261(50.8%) | 54(59.3%) | 45(50.6%) |
| **T2** | 32(12.5%) | 34(13.2%) | 66(12.9%) | 13(14.3%) | 10(11.2%) |
| **T3** | 101(39.5%) | 74(28.8%) | 175(34.1%) | 22(24.2%) | 33(37.1%) |
| **T4** | 6(2.3%) | 5(2.0%) | 11(2.1%) | 2(2.2%) | 1(1.1%) |
| **M stage** |  |  |  |  |  |
| **M0** | 197(77.0%) | 213(82.9%) | 410(79.9%) | 81(89.0%) | 33(37.1%) |
| **M1** | 43(16.8%) | 34(13.2%) | 77(15.0%) | 9(9.9%) | 11(12.4%) |
| **Mx** | 16(6.2%) | 10(3.9%) | 26(5.1%) | 1(1.1%) | 45(50.5%) |
| **N stage** |  |  |  |  |  |
| **N0** | 122(47.7%) | 112(43.6%) | 234(45.6%) | 79(86.8%) | 14(15.7%) |
| **N1** | 9(3.5%) | 5(1.9%) | 14(2.7%) | 2(2.2%) |  |
| **Nx** | 125(48.8%) | 140(54.5%) | 265(51.7%) | 10(11.0%) | 75(84.3%) |
| **Survival time(years)** | 3.68(0.01-12.4) | 3.66(0.02-10.8) | 3.33(0.01-12.43) | 4.8(0.01-6.2) | 4.8(0.13-2.3) |
| **Survival status** |  |  |  |  |  |
| **Live** | 171(66.8%) | 173(67.3%) | 344(67.1%) | 61(67.0%) | 77(86.5%) |
| **Dead** | 85(33.2%) | 84(32.7%) | 169(32.9%) | 30(33.0%) | 12(13.5%) |

**Supplementary Table 1**
